# Circulating Angiopoietin-2 and Its Soluble Receptor Tie-2 Concentrations Are Related to Renal Function in Two Population-Based Cohorts

**DOI:** 10.1371/journal.pone.0166492

**Published:** 2016-11-28

**Authors:** Anna Hennings, Anke Hannemann, Rainer Rettig, Marcus Dörr, Matthias Nauck, Henry Völzke, Markus M. Lerch, Wolfgang Lieb, Nele Friedrich

**Affiliations:** 1 Institute of Clinical Chemistry and Laboratory Medicine, University Medicine Greifswald, Greifswald, Germany; 2 Institute of Physiology, University Medicine Greifswald, Greifswald, Germany; 3 Department of Internal Medicine B, University Medicine Greifswald, Greifswald, Germany; 4 DZHK (German Centre for Cardiovascular Research), partner site Greifswald, Germany; 5 Institute for Community Medicine, University Medicine Greifswald, Greifswald, Germany; 6 DZD (German Centre for Diabetes Research), partner site Greifswald, Greifswald, Germany; 7 Department of Internal Medicine A, University Medicine Greifswald, Greifswald, Germany; 8 Institute of Epidemiology, Christian-Albrechts University of Kiel, Kiel, Germany; 9 Research Centre for Prevention and Health, Capital Region of Denmark, Glostrup, Denmark; The University of Tokyo, JAPAN

## Abstract

**Background:**

An intact angiopoietin/Tie-2 ligand receptor system is indispensable for life. High circulating angiopoietin-2 (Ang-2) concentrations are strongly associated with kidney disease involving the progressive loss of glomerular filtration. The aim of our study was to investigate the associations between renal function and serum Ang-2 or serum Tie-2 concentrations in the general population.

**Methods:**

Data of 3081 and 4088 subjects from two population-based studies, the Study of Health in Pomerania (SHIP-1) and SHIP-Trend, were used. Renal function was assessed by serum creatinine, cystatin C concentration, creatinine-based estimated glomerular filtration rate [eGFR(crea)], cystatin C-based eGFR [eGFR(cys)] and urinary albumin-to-creatinine ratio (uACR). Analyses of variance and linear regression models were calculated.

**Results:**

In both cohorts, strong positive associations between serum cystatin C concentrations and serum Ang-2 or Tie-2 concentrations as well as inverse associations between eGFR(cys) and serum Ang-2 or Tie-2 concentrations were found. These relations were also present in a subpopulation without hypertension or diabetes mellitus type 2. Furthermore, we detected weak U-shaped associations between serum creatinine concentrations or eGFR(crea) and serum Ang-2 concentrations. With respect to uACR a strong positive association with serum Ang-2 concentrations was revealed.

**Conclusion:**

Serum Ang-2 concentrations are strongly associated with sensitive parameters of renal impairment like serum cystatin C, uACR and eGFR(cys). These findings persisted even after exclusion of subjects with hypertension or diabetes mellitus type 2, conditions that predispose to chronic renal disease and are associated with increased Ang-2 concentrations. Interestingly, we did not detect the same strong relations between serum creatinine and eGFR(crea) with serum Ang-2 concentration. Additionally, significant association of serum Tie-2 concentrations with cystatin C and eGFR(cys) were detected.

## Introduction

The angiopoietin/Tie-2 ligand receptor system is an important regulator of the vascular integrity and endothelial cell homeostasis [1]. The best known ligands of the system are angiopoietin 1 (Ang-1) and angiopoietin 2 (Ang-2), two endothelial growth factors [1] that bind to the same extracellular domain of the Tie-2 receptor, but evoke opposing biological effects [2]. After phosphorylation of the receptor, Ang-1 is involved in vessel integrity, cell-cell stabilization and depression of inflammatory genes [3]. Ang-2, on the other hand, acts as an antagonist by disrupting the protective signaling of Ang-1/Tie-2. The peptide promotes inflammation, weakens the endothelial barrier function and enhances the endothelial responsiveness to stimulation by inflammatory and angiogenic cytokines, like vascular endothelial growth factor (VEGF) or tumor necrosis factor (TNF)[1]. Ang-1 is produced and constitutively secreted by vascular smooth muscle cells and pericytes. Ang-2 is synthesized by endothelial cells and stored in Weibel-Palade bodies[1, 4]. Physical damage, hypoxia, altered shear stress or soluble factors like VEGF and TNFα are stimuli for the rapid release of Ang-2 from Weibel-Palade bodies [1, 4]. Accordingly, Ang-2 appears to function as an autocrine negative regulator of the resting endothelium [1, 4, 5].

During the last decade, increased circulating Ang-2 concentrations have been reported in a broad range of diseases including diabetes mellitus [6], sepsis [7], acute pancreatitis [8], cardiovascular diseases [9] and critical illness [10]. Particularly strong associations have been detected with chronic kidney disease (CKD), during haemodialysis, or after kidney transplantation [9].

An intact angiopoietin/Tie-2 ligand receptor system is indispensable for life. In animal experiments, deletion of the Ang-1 or the Tie-2 gene is embryonically lethal [11–13]. An animal study [14], with induced podocyte-specific Ang-2 overexpressing in mice showed that increased glomerular expression of Ang-2 was accompanied by enhanced glomerular apoptosis. The overexpression of Ang-2 was further associated with increased proteinuria as well as with the downregulation of VEGF-A and nephrin, critical players in the maintenance of the filtration barrier. In patients with CKD, dialysis patients or renal transplant recipients, the progressive decline in glomerular filtration rate (GFR) is often associated with a significant elevation in serum Ang-2 concentrations [9]. In a further study in CKD patients, the estimated GFR (eGFR) was inversely related to circulating Ang-2 concentrations and Ang-2 concentrations rose with advancing CKD stages [15].

Although several clinical studies [9, 11, 15, 16] investigated the association between clinical renal diseases and circulating Ang-2 concentrations, there are currently no studies addressing the relation between renal function and circulating Ang-2 concentrations in the general population. We therefore investigated the associations of functional parameters of the kidney (serum creatinine, cystatin C concentrations, urinary albumin-to-creatinine ratio (uACR) or creatinine and cystatin C-based eGFR) with serum Ang-2 concentrations as well as with serum Tie-2 concentrations in the population-based Study of Health in Pomerania (SHIP) consisting of two independent cohorts (SHIP-1 and SHIP-Trend) with a study population of more than 7000 subjects.

## Material and Methods

### Study population

SHIP and SHIP-Trend are independent, population-based cohort studies in the northeast of Germany. The study design and sampling methods have been previously described [17]. In short, 4308 men and women from a representative population sample of 7008 subjects participated in the baseline examinations of the SHIP cohort (SHIP-0: October 1997 to May 2001). Of these, 3300 subjects participated in the first five-year follow-up examinations (SHIP-1: March 2002 to July 2006). An additional 4420 men and women from a representative sample of 8016 adults participated in the baseline examinations of the SHIP-Trend cohort (SHIP-Trend: September 2008 to September 2012). As the study region of the two cohorts is largely similar, participation in the SHIP cohort was an exclusion criterion for participation in SHIP-Trend. All SHIP and SHIP-Trend participants gave written informed consent. Both studies follow the recommendations of the Declaration of Helsinki and were approved by the ethics committee of the University of Greifswald. Further, both studies were reviewed by an external scientific review board.

For the present analyses data from SHIP-1 and SHIP-Trend were used as in these studies, serum Ang-2 and serum Tie-2 concentrations were measured. SHIP-Trend data were used to validate the findings from SHIP-1.

Of the 3300 SHIP-1 participants, 203 subjects were excluded due to the presence of at least one of the following conditions (overlap exists): missing or extreme values (>99 percentile to minimize the influence of outliers on least squares analyses) for serum Ang-2 concentration (n = 70), serum Tie-2 concentration (n = 66) or renal parameters (n = 120), or a creatinine-based eGFR <30 ml/min/1.73m^2^ (n = 18). Furthermore, 16 subjects with missing values for confounding factors were excluded. The final SHIP-1 study sample comprised 3081 individuals. Of the 4420 SHIP-Trend participants, 295 subjects were excluded due to the presence of at least one of the following conditions (overlap exists): missing or extreme values (>99 percentile to minimize the influence of outliers on least squares analyses) for serum Ang-2 concentration (n = 122), serum Tie-2 concentration (n = 118) or renal parameters (n = 154), or an creatinine-based eGFR <30 ml/min/1.73m^2^ (n = 12). Furthermore, 37 subjects with missing values for confounding factors were excluded. The final SHIP-Trend study sample comprised 4088 subjects.

### Measurements

For both cohorts, information on age, sex, socio-demographic characteristics and medical histories were gained by computer-aided personal interviews. Smoking status was assessed by self-report. Waist circumference was measured to the nearest 0.1 cm using an inelastic tape midway between the lower rib margin and the iliac crest in the horizontal plane, with the subject standing comfortably with weight distributed evenly on both feet. The measurement was taken at the level of the narrowest part of the waist. After a 5 minute resting period, systolic and diastolic blood pressure was measured three times on the right arm of seated subjects using a digital blood pressure monitor (HEM-705CP, Omron Corporation, Tokyo, Japan) with each reading being followed by a further resting period of 3 minutes. The last two readings were averaged to obtain the mean diastolic and systolic blood pressure. Increased blood pressure was defined as a systolic blood pressure of ≥140 mmHg or a diastolic blood pressure of ≥90 mmHg. Hypertension was defined by either an increased blood pressure or the self-reported use of antihypertensive medication. The definition of diabetes mellitus was based on HbA1c > 6.5% or self-reported use of antidiabetic medication [anatomic, therapeutic and chemical (ATC) code: A10] in the last 7 days prior to the examination.

Blood samples were drawn from the cubital vein in the supine position and serum aliquots were prepared for immediate analysis and for storage at -80°C. In SHIP-1, serum creatinine concentrations were determined with a modified kinetic Jaffé method (Siemens Dimension RxL; Siemens Healthcare Diagnostics, Eschborn, Germany) with an analytical sensitivity of 0.10–20.0 mg/dl. Serum cystatin C concentrations were measured with a nephelometric assay (BN ProSpec; Siemens Healthcare Diagnostics, Eschborn Germany) with an analytical sensitivity of 0.23–8.0 mg/l. In SHIP-Trend, serum creatinine concentrations were determined with a modified kinetic Jaffé method with a sensitivity of 0.14–20.2 mg/dl (Dimension VISTA, Siemens Healthcare Diagnostics, Eschborn, Germany). Serum cystatin C concentrations were measured using a nephelometric assay (Dimension VISTA, Siemens Healthcare Diagnostics, Eschborn, Germany) with a functional sensitivity of 0.05 mg/l. The creatinine-based eGFR was calculated using the four-variable Modification of Diet in Renal Disease (MDRD) study equation: eGFR(crea) = 186.3 × serum creatinine^-1.154^ × age^-0.203^ × (0.742 if female) [18, 19]. Furthermore, cystatin C-based eGFR was calculated using the CKD-EPI cystatin C equation: eGFR(cys) = 133 × min(serum cystatin C / 0.8, 1)^-0.499^ × max(serum cystatin C / 0.8, 1)^-1.328^ × 0.996^age^ [× 0.932 if female][20]. A comparison of both eGFRs revealed a moderate correlation between both measurements (Figure A in S1 File). Serum Ang-2 and serum Tie-2 concentrations were measured by solid-phase enzyme-linked immunosorbent assays (R&D systems) in both studies, although using different assay charges. The minimum detectable concentrations were 1.20 pg/ml and 0.001 ng/ml for serum Ang-2 and serum Tie-2 concentrations, respectively. In SHIP-1, the inter-assay coefficients of variation were 13.3% and 10.6% for Ang-2 as well as 9.7% and 6.8% for Tie-2, for median and high serum biomarker concentrations, respectively. In SHIP-Trend the inter-assay coefficients of variation were 15.4% and 9.8% for Ang-2 and 10.5% and 5.7% for Tie-2, for median and high serum biomarker concentrations, respectively. Serum total cholesterol concentrations were determined on the Dimension RxL (Siemens Healthcare Diagnostics, Eschborn, Germany) in SHIP-1 and on the Dimension VISTA (Siemens Healthcare Diagnostics, Eschborn, Germany) in SHIP-Trend.

Urine samples were collected between 7.00 a.m. and 6.00 p.m. The urinary creatinine concentration was measured with a modified kinetic Jaffé reaction in SHIP-1 (Siemens Dimension RxL, Siemens Healthcare Diagnostics, Eschborn, Germany) and with a photometric reaction in SHIP-Trend (Dimension VISTA, Siemens Healthcare Diagnostics, Eschborn, Germany). The urinary albumin concentration was measured with nephelometric assays in SHIP-1 and SHIP-Trend (SHIP-1: BN ProSpec Analyzer, Dade Behring, Deerfield, IL, USA; SHIP-Trend: Dimension VISTA, Siemens Healthcare Diagnostics, Eschborn, Germany). The uACR was calculated using the following equation: uACR (mg/mmol) = urinary albumin concentration (mg/L)/urinary creatinine concentration (mmol/L).

### Statistical analyses

Continuous data are expressed as median (25^th^; 75^th^ quartile). Nominal data are expressed as percentage. For bivariate comparisons the Kruskal-Wallis test (continuous data) or χ^2^-test (nominal data) were used to compare SHIP-1 and SHIP-Trend. Analyses of variance (ANOVA) and multivariable linear regression models were performed to estimate the independent associations of serum creatinine and eGFR(crea), serum cystatin C and eGFR(cys) as well as uACR (exposure variables) as either categorical or continuous variables with serum Ang-2 or serum Tie-2 concentrations (outcome variables). Serum Ang-2 and uACR concentrations were log-transformed to approximate a normal distribution. In ANOVA, exposure variables were categorized into four groups according to their sex-specific quartiles. To detect possible nonlinear associations, linear regression models with restricted cubic splines with three knots pre-specified located at the 5th, 50th and 95th percentile as recommended by Stone and Koo [21] were compared by likelihood ratio test to the fit of a linear model. All analyses were performed for the whole population of SHIP-1 and SHIP-Trend as well as in subsets, excluding individuals with hypertension or diabetes mellitus type 2 [SHIP-1: n = 1663 (53.4%); SHIP-Trend: n = 1660 (52.2%)]. ANOVA models were adjusted for age, sex and waist circumference. Linear regression models were additionally adjusted for smoking, total cholesterol concentration, systolic and diastolic blood pressure as well as in the whole population for diabetes mellitus type 2 and use of antihypertensive medication. In 555 SHIP-1 and 1155 SHIP-Trend subjects, urinary albumin concentrations were below the limit of detection; therefore, analyses regarding uACR were performed in subpopulations. A p-value of < 0.05 was considered statistically significant. Statistical analyses were performed with SAS 9.4 (SAS Institute Inc., Cary, NC, USA).

## Results

**Fig 1:**
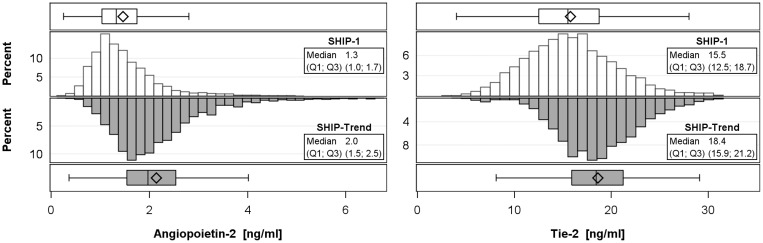
Histograms and boxplots of serum angiopoietin-2 and Tie-2 receptor concentrations by study sample (SHIP-1 and SHIP-Trend).

General characteristics of the SHIP-1 (n = 3081) and the SHIP-Trend sample (n = 4088) are shown in Table 1. SHIP-1 subjects were older, more often never smokers and had higher systolic and diastolic blood pressure than SHIP-Trend participants. Regarding renal function, SHIP-1 participants had higher serum creatinine and cystatin C concentrations and consequently lower eGFR than SHIP-Trend subjects. Median serum concentrations of Ang-2 and Tie-2 were lower in SHIP-1 than in SHIP-Trend (SHIP-1: Ang-2: 1.3 ng/ml; Tie-2: 15.5 ng/ml and SHIP-Trend: Ang-2: 2.0 ng/ml; Tie-2: 21.2 ng/ml) (Fig 1).

**Table 1:** Baseline characteristics stratified by study population.

Characteristics	SHIP-1 (n = 3081)	SHIP-Trend (n = 4088)	P*
Age (years)	55 (42; 66)	53 (40; 64)	<.01
Men (%)	48	48	0.86
Smoking (%)			<.01
never smokers	42	37	
former smokers	32	37	
current smokers	26	27	
Waist circumference (cm)	92 (83; 102)	91 (80; 101)	<.01
Systolic blood pressure (mmHg)	131 (119; 144)	127 (115; 140)	<.01
Diastolic blood pressure (mmHg)	81 (74; 88)	77 (71; 84)	<.01
Total Cholesterol (mmol/l)	5.5 (4.7; 6.3)	5.4 (4.7; 6.2)	<.01
Hypertension (%)	51	48	<.01
Diabetes mellitus type 2 (%)	11	9	0.02
Creatinine (μmol/l)	78 (68; 89)	77 (67; 88)	<.01
eGFR(crea) (ml/min/1.73m²)	83.4 (71.8; 96.5)	86.5 (74.5; 99.9)	<.01
Cystatin C (mg/l)	0.82 (0.73; 0.94)	0.71 (0.64; 0.80)	<.01
eGFR(cys) (ml/min/1.73m²)	99.4 (80.3; 112.0)	111.4 (99.4; 121.1)	<.01
uACR (mg/mmol)**	1.00 (0.56; 2.27)	0.95 (0.60; 1.91)	0.87

eGFR = estimated glomerular filtration rate based on serum creatinine [eGFR(crea)] or cystain C [eGFR(cys)]; uACR = urinary albumin-to-creatinine ratio. Data are expressed as median (25^th^ percentile; 75^th^ percentile); nominal data are given as percentages.

* χ^2^-test (nominal data) or Mann-Whitney test (interval data).

** uACR were only available in subpopulations (SHIP-1: n = 2518, SHIP-Trend: n = 2924).

**Fig 2:**
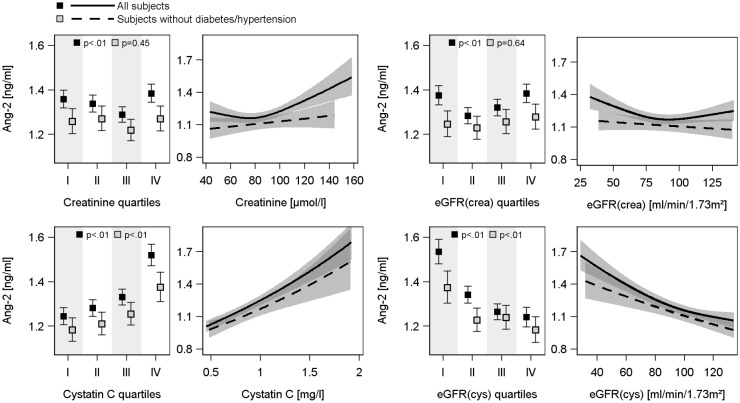
Associations between serum creatinine concentration, creatinine-based estimated glomerular filtration rate [eGFR(crea)], serum cystatin C concentration or cystatin C-based eGFR [eGFR(cys)] and serum angiopoietin-2 (Ang-2) concentration in the SHIP-1 population. For each exposure left side: Estimated mean serum Ang-2 with 95% confidence intervals (CI) by sex-specific quartiles of exposure calculated by analysis of variance adjusted for age, sex and waist circumference. Right side: linear regression line with 95% CI (grey shaded area). Linear regression models with restricted cubic splines were adjusted for age, sex, waist circumference, smoking, total cholesterol, systolic and diastolic blood pressure and additionally in the whole population for diabetes mellitus type 2 and use of antihypertensive medication.

**Fig 3:**
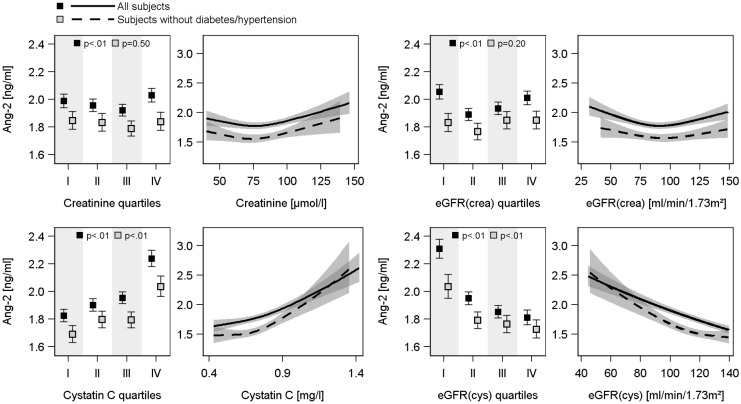
Associations between serum creatinine concentration, creatinine-based estimated glomerular filtration rate [eGFR(crea)], serum cystatin C concentration or cystatin C-based eGFR [eGFR(cys)] and serum angiopoietin-2 (Ang-2) concentration in the SHIP-Trend population. For each exposure left side: Estimated mean serum Ang-2 with 95% confidence intervals (CI) by sex-specific quartiles of exposure calculated by analysis of variance adjusted for age, sex and waist circumference. Right side: linear regression line with 95% CI (grey shaded area). Linear regression models with restricted cubic splines were adjusted for age, sex, waist circumference, smoking, total cholesterol, systolic and diastolic blood pressure and additionally in the whole population for diabetes mellitus type 2 and use of antihypertensive medication.

**Fig 4:**
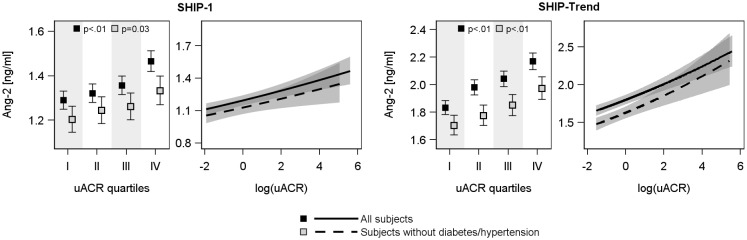
Associations between urinary albumin-to-creatinine ratio (uACR) and serum angiopoietin-2 concentration in SHIP-1 (left side) and SHIP-Trend (right side). For each study population left side: Estimated mean serum Ang-2 with 95% confidence intervals by sex-specific quartiles of uACR calculated by analysis of variance adjusted for age, sex and waist circumference. Right side: linear regression line with 95% confidence intervals (grey shaded area). Linear regression models with restricted cubic splines were adjusted for age, sex, waist circumference, smoking, total cholesterol, systolic and diastolic blood pressure and additionally in the whole population for diabetes mellitus type 2 and use of antihypertensive medication.

ANOVA (Figs 2 and 3) revealed U-shaped associations of serum creatinine and eGFR(crea) with serum Ang-2 concentrations, with the lowest Ang-2 values in the 2^nd^ or 3^th^ quartile of creatinine or eGFR(crea). However, these associations disappeared after the exclusion of subjects with hypertension or diabetes mellitus type 2. Serum cystatin C concentration was positively and eGFR(cys) was negatively associated with serum Ang-2 and Tie-2 concentrations in both cohorts (Figs 2, 3 and figure B in S1 File). These relations were also present in the subsample excluding subjects with hypertension or diabetes mellitus type 2. With respect to uACR positive associations to serum Ang-2 concentrations were found in both study populations even after exclusion of diseased subjects (Fig 4), whereas the associations to serum Tie-2 concentrations were lost after exclusion of subjects with hypertension or diabetes mellitus type 2 (Figure C in S1 File).

In concordance with the ANOVA, linear regression analyses (Figs 2 and 3, Table 2) using continuous exposure variables confirmed the U-shaped associations of serum creatinine concentrations or eGFR(crea) with serum Ang-2 concentrations in both cohorts. The lowest serum Ang-2 concentrations were identified for serum creatinine concentrations of 80 μmol/l or eGFR(crea) values of 80 ml/min/1.73m^2^, respectively. Furthermore, the strong relations between serum cystatin C or eGFR(cys) and serum Ang-2 or Tie-2 concentrations were confirmed (Figs 2, 3 and figure B in S1 File, Table 2). The exclusion of subjects with hypertension or diabetes mellitus type 2 eliminated the associations between serum creatinine or the eGFR(crea) and serum Ang-2 concentrations but did not substantially affect the findings for serum cystatin C and eGFR(cys) (Figs 2 and 3, Table 2). Moreover, the additional adjustment for high-sensitive C-reactive protein (hsCRP) as marker of inflammation did not change the results (data not shown). With respect to uACR, linear regression confirmed the positive associations to serum Ang-2 concentrations and further revealed slightly positives associations with serum Tie-2 concentrations (Fig 4 and figure C in S1 File, Table 2).

**Table 2:** Associations between markers of renal function and serum angiopoietin-2 concentration or serum Tie-2 concentration.

	Angiopoietin-2			Tie-2			Angiopoietin-2			Tie-2		
	Beta	Stderr	P	Beta	Stderr	P	Beta	Stderr	P	Beta	Stderr	P
**SHIP-1**												
Creatinine	-1.88E^-03^	1.16E^-03^	0.10	-9.80E^-04^	6.02E^-03^	0.87	1.09E^-03^	8.80E^-04^	0.22	-3.80E^-03^	1.03E^-02^	0.71
Creatinine'	1.67E^-06^	5.04E^-07^	<.01				-	-	-	-	-	-
eGFR(crea)	-3.53E^-03^	9.95E^-04^	<.01	-1.15E^-03^	5.21E^-03^	0.83	-7.05E^-04^	6.59E^-04^	0.29	3.05E^-03^	7.71E^-03^	0.69
eGFR(crea)'	9.76E^-07^	3.09E^-07^	<.01	-	-	-	-	-	-	-	-	-
Cystatin C	0.39	0.04	<.01	5.84	1.26	<.01	0.35	0.08	<.01	3.67	0.97	<.01
Cystatin C'	-	-	-	-15.03	4.87	<.01	-	-	-	-	-	-
eGFR(cys)	-5.53E^-03^	8.00E^-04^	<.01	-9.24E^-03^	9.54E^-03^	0.33	-3.73E^-03^	8.44E^-04^	<.01	-3.78E^-02^	9.88E^-03^	<.01
eGFR(cys)'	3.58E^-07^	1.74E^-07^	0.04	-5.08E^-06^	2.07E^-06^	0.01	-	-	-			
log(uACR)	3.67E^-02^	7.10E-03	<.01	0.20	0.08	0.02	3.51E^-02^	1.23E^-02^	<.01	0.28	0.15	0.05
**SHIP-Trend**												
Creatinine	-2.39E^-03^	9.76E^-04^	0.01	9.99E^-03^	4.80E^-03^	0.04	-2.97E^-03^	1.54E^-03^	0.05	1.31E^-02^	7.47E^-03^	0.08
Creatinine'	1.64E^-06^	4.41E^-07^	<.01	-	-	-	2.37E^-06^	9.18E^-07^	0.01	-	-	-
eGFR(crea)	-3.48E^-03^	8.03E^-04^	<.01	-7.69E^-03^	3.78E^-03^	0.04	-2.42E^-03^	1.22E^-03^	0.05	-7.02E^-03^	5.16E^-03^	0.17
eGFR(crea)'	1.12E^-06^	2.40E^-07^	<.01	-	-	-	9.51E^-07^	4.22E^-07^	0.02	-	-	-
Cystatin C	0.25	0.12	0.03	1.18	0.54	0.03	0.05	0.20	0.81	2.53	0.90	<.01
Cystatin C'	1.67	0.86	0.05	-	-	-	5.81	2.27	0.01	-	-	-
eGFR(cys)	-4.79E^-03^	4.99E^-04^	<.01	-1.20E^-02^	5.53E^-03^	0.03	-7.76E^-03^	1.29E^-03^	<.01	-2.50E^-02^	9.19E^-03^	0.01
eGFR(cys)'	-	-	-	-	-	-	1.45E^-06^	5.98E^-07^	0.02	-	-	-
log(uACR)	5.47E-02	7.10E-03	<.01	-0.60	0.22	<.01	6.46E^-02^	1.26E^-02^	<.01	-1.01	0.39	<.01
log(uACR)'	-	-	-	9.52E^-02^	3.62E^-02^	<.01	-	-	-	0.23	0.09	0.01

eGFR = estimated glomerular filtration rate based on either serum creatinine [eGFR(crea)] or serum cystatin C concentration [eGFR(cys)]. All models were adjusted for age, sex, waist circumference, smoking, total cholesterol, systolic and diastolic blood pressure and additionally in the whole population for diabetes mellitus type 2 and use of antihypertensive or antidiabetic medication. Creatinine', eGFR' and cystatin C' represent spline components, for more detail see method section.

## Discussion

Our data show U-shaped associations between serum creatinine or eGFR(crea) and serum Ang-2 concentrations in two independent cohorts. These associations did not persist after exclusion of subjects with hypertension or diabetes mellitus type 2. Furthermore, serum cystatin C concentrations were positively and eGFR(cys) was negatively associated with the serum Ang-2 concentration. However, these associations were generally stronger than those between serum creatinine or eGFR(crea) and serum Ang-2 concentrations and persisted after exclusion of subjects with hypertension or diabetes mellitus type 2. Serum Tie-2 concentrations reveal only few significant correlations with serum cystatin C concentrations and eGFR(cys). Moreover, out data revealed a stable and strong positive association between uACR and serum Ang-2 concentrations in both investigated study populations.

### Association of eGFR, serum cystatin C and uACR with serum Ang-2 concentrations

Previous studies [9, 11, 16] have reported associations between kidney disease, involving endothelial dysfunction, and circulating Ang-2 concentrations. David and Kümpers [16] found an inverse correlation between inulin-based GFR and serum Ang-2 concentration in patients with CKD stages 1–4 and reported a continuous increase in serum Ang-2 concentration with deteriorating renal function. Likewise an elevation of serum Ang-2 concentrations was noted in healthy kidney donors following unilateral nephrectomy. After the sudden loss of one kidney, the decline in cystatin C-based eGFR was paralleled by an increase in circulating Ang-2 concentration [16]. In addition, elevated circulating Ang-2 concentration was observed in CKD patients on renal replacement therapy and was neither affected by the method nor by the frequency of dialysis treatment [9]. After successful kidney transplantation, circulating Ang-2 concentration normalized [9]. In the present study, an inverse association between eGFR(cys) and serum Ang-2 concentration was detected in SHIP-1 and the results were validated in the replication cohort SHIP-Trend. Both population-based cohorts include large numbers of healthy volunteers [subjects with eGFR(crea) >60 ml/min/1.73m^2^: SHIP-1 n = 2814 (91.3%); SHIP-Trend n = 3829 (93.7%)], thus confirming that the above-mentioned association also exists outside the area of overt renal disease. The association remained statistically significant after exclusion of subjects with hypertension or diabetes mellitus type 2. Interestingly, we did not detect a strong inverse relation between eGFR(crea) and serum Ang-2 concentration.

Cystatin C is a nonglucosylated, low molecular weight protein, which is constantly produced by all nucleated cells. After free filtration through the glomeruli, it is reabsorbed and catabolized in the proximal tubule [22]. Like creatinine, cystatin C is used as a biomarker of kidney function, but it is less dependent on age, sex and muscle mass [23, 24]. Moreover, cystatin C has no “blind area” as creatinine, in which the serum creatinine concentrations only increase significant beyond reduced kidney function >50% [25]. Therefore, the serum cystatin C concentration, is supposed to be a more sensitive marker of GFR than the serum creatinine concentration [26].

The uACR is an established marker for glomerular injury and renal dysfunction, vascular damage and systemic inflammation [27–29]. Chang and Lai [27] investigated the relation between albuminuria and plasma Ang-2 concentrations in 416 patients with CKD stages 3 to 5 and showed a positive association between uACR and plasma Ang-2 concentration. However, uACR and plasma levels of Tie-2 showed no relation. In the present study, we confirmed these findings and observed a strong positive association of uACR with serum Ang-2 concentration in a population-based setting. This association, similar to the relation of serum Ang-2 concentrations with serum cystatin C and eGFR(cys), persists even after the exclusion of subjects with hypertension or diabetes mellitus type 2. The latter conditions are known to be associated with elevated serum Ang-2 and cystatin C concentrations [30, 31]. Although the detailed underlying pathophysiology is unknown, the found associations strengthen the reported association to cystatin C and suggest that cystatin C or uACR and Ang-2 are either associated with each other or influenced by the same (renal) events. We note that, in contrast, the associations of serum creatinine concentrations and eGFR(crea) with serum Ang-2 concentrations got lost or weaker in the healthy subpopulation.

Ang-2 is supposed to reflect the amount of endothelial activation [32] and to predict the severity and outcome in critically ill patients or of illnesses like acute pancreatitis [32], CKD [15], or after cardiac surgery [33], in early stages of the medical conditions. These predictions were often linked to an deterioration of renal function [15, 32, 33]. Taking into account the differences in the present study between serum creatinine/eGFR(crea) and serum cystatin C/eGFR(cys) and the assumption that serum cystatin C or the eGFR(cys) are more sensitive and earlier markers of renal dysfunction or damage [16, 26, 34], one possible conclusion might be that serum cystatin C concentrations, eGFR(cys) and serum Ang-2 concentrations might be reacting sooner towards renal impairment with potential endothelial activation than serum creatinine concentrations. However the causal relationship is still obscure and further studies are necessary to investigate the exact mechanisms linking cystatin C, uACR and eGFR(cys) with increased Ang-2 concentration.

### Weibel-Palade bodies and the release of Ang-2

The renal endothelium appears to be an extensive source of Ang-2 [3, 16]. Ang-2 is stored in Weibel-Palade bodies and rapidly released upon several triggers mirroring the activation of the endothelium. The only known inhibitor of the Weibel-Palade bodies is nitric oxide (NO), which is decreased in CKD patients in the presence of high asymmetric dimethylarginine (ADMA), an endogenous nitric oxide synthase inhibitor [9, 35]. A Chinese study [36] found that increased circulating ADMA concentrations were associated with elevated circulating cystatin C concentrations in patients with coronary artery disease. The authors [36] attributed this association to a reduced renal function in their coronary artery disease patients. Additionally, elevated circulating ADMA concentrations and thus a reduced NO production are early forerunners of CKD, and may precede other diagnostic signs like the reduction of GFR [37]. Furthermore, cystatin C was found to be a marker not only of renal function but also of inflammation, as associations between cystatin C and proinflammatory cytokines like TNFα were detected [38]. Interestingly, serum creatinine concentrations and eGFR showed no association with this cytokine [38]. TNFα is a trigger for exocytosis of Weibel-Palade bodies [1, 39, 40]. Thus, all three parameters, i.e., circulating Ang-2, cystatin C and ADMA concentrations, rise in parallel with early renal impairment and endothelial dysfunction. In order to consider the potential influence of inflammation we additionally adjusted our linear regression for the hsCRP concentration. The adjustment did not weaken our associations between serum Ang-2 or serum Tie-2 concentration with the investigated exposure variables. However, as we are unable to assess longitudinal or causal relations we cannot fully exclude an influence of inflammation on the observed association. This is in particular the case, as the hsCRP concentration was measured at a single occasion.

### Strengths and limitations

The key strength of our study is the population-based design covering two large and independent cohorts with a total of 7169 volunteer subjects. Another strength is the high grade quality assurance of the laboratory methods and the performance of the measurements by skilled technicians following a standardized protocol. A limitation emerges from the cross-sectional study design, and the missing repeated measurements of serum Ang-2 or serum Tie-2 concentration, which prohibit to assess causal relations. Therefore, further analyses investigating whether changes in renal function over time would lead to subsequent changes in serum Ang-2 concentrations are needed to assess to what extent the latter may serve as prognostic biomarkers.

## Conclusion

In two population-based cohorts comprising more than 7000 volunteers we found that, even after exclusion of subjects with conditions that predispose to chronic renal disease such as hypertension or diabetes mellitus type 2, serum Ang-2 concentration associate with the most sensitive parameters of renal impairment, serum cystatin C, uACR and eGFR(cys). Interestingly, we did not detect a comparable strong relation between serum creatinine and eGFR(crea) with the serum Ang-2 concentration. Additionally significant association of serum Tie-2 concentrations with cystatin C and eGFR(cys) were detected. Future prospective trials will have to elucidate whether serum Ang-2 concentrations alone or in combination with serum cystatin C concentrations might improve the (early) diagnosis of kidney damage or serve as a prognostic marker for chronic renal failure.

## Supporting Information

S1 File **Figure A**. **Scatterplot of creatinine-based [eGFR(crea)] versus cystatin C-based estimated glomerular filtration rate [eGFR(cys)]** in both populations. Pearson correlation is given. (Figure A in S1 File) **Figure B. Associations between serum creatinine concentration, creatinine-based estimated glomerular filtration rate [eGFR(crea)], serum cystatin C concentration or cystatin C-based eGFR [eGFR(cys)] and serum Tie-2 receptor concentration** in the SHIP-1 (upper part) and SHIP-Trend (lower part) population. For each exposure left side: Estimated mean serum Tie-2 receptor with 95% confidence intervals (CI) by sex-specific quartiles of exposure calculated by analysis of variance adjusted for age, sex and waist circumference. Right side: linear regression line with 95% CI (grey shaded area). Linear regression models with restricted cubic splines were adjusted for age, sex, waist circumference, smoking, total cholesterol, systolic and diastolic blood pressure and additionally in the whole population for diabetes mellitus type 2 and use of antihypertensive medication. (Figure B in S1 File) **Figure C. Associations between urinary albumin-to-creatinine ratio (uACR) and serum Tie-2 receptor concentration** in SHIP-1 (left side) and SHIP-Trend (right side). For each study population left side: Estimated mean serum Tie-2 receptor with 95% confidence intervals (CI) by sex-specific quartiles of uACR calculated by analysis of variance adjusted for age, sex and waist circumference. Right side: linear regression line with 95% CI (grey shaded area). Linear regression models with restricted cubic splines were adjusted for age, sex, waist circumference, smoking, total cholesterol, systolic and diastolic blood pressure and additionally in the whole population for diabetes mellitus type 2 and use of antihypertensive medication. (Figure C in S1 File)
(PDF)
