# Correction: Circulating Angiopoietin-2 and Its Soluble Receptor Tie-2 Concentrations Are Related to Renal Function in Two Population-Based Cohorts

**DOI:** 10.1371/journal.pone.0176477

**Published:** 2017-04-19

**Authors:** The PLOS ONE Staff

The first row of Table 2 is missing. Please view the corrected table below. The publisher apologizes for the error.

**Table 2:** Associations between markers of renal function and serum angiopoietin-2 concentration or serum Tie-2 concentration.

	All subjects						Subjects without hypertension or diabetes mellitus					
	Angiopoietin-2			Tie-2			Angiopoietin-2			Tie-2		
	Beta	Stderr	P	Beta	Stderr	P	Beta	Stderr	P	Beta	Stderr	P
**SHIP-1**												
Creatinine	-1.88E^-03^	1.16E^-03^	0.10	-9.80E^-04^	6.02E^-03^	0.87	1.09E^-03^	8.80E^-04^	0.22	-3.80E^-03^	1.03E^-02^	0.71
Creatinine'	1.67E^-06^	5.04E^-07^	<.01				-	-	-	-	-	-
eGFR(crea)	-3.53E^-03^	9.95E^-04^	<.01	-1.15E^-03^	5.21E^-03^	0.83	-7.05E^-04^	6.59E^-04^	0.29	3.05E^-03^	7.71E^-03^	0.69
eGFR(crea)'	9.76E^-07^	3.09E^-07^	<.01	-	-	-	-	-	-	-	-	-
Cystatin C	0.39	0.04	<.01	5.84	1.26	<.01	0.35	0.08	<.01	3.67	0.97	<.01
Cystatin C'	-	-	-	-15.03	4.87	<.01	-	-	-	-	-	-
eGFR(cys)	-5.53E^-03^	8.00E^-04^	<.01	-9.24E^-03^	9.54E^-03^	0.33	-3.73E^-03^	8.44E^-04^	<.01	-3.78E^-02^	9.88E^-03^	<.01
eGFR(cys)'	3.58E^-07^	1.74E^-07^	0.04	-5.08E^-06^	2.07E^-06^	0.01	-	-	-			
log(uACR)	3.67E^-02^	7.10E-03	<.01	0.20	0.08	0.02	3.51E^-02^	1.23E^-02^	<.01	0.28	0.15	0.05
**SHIP-Trend**												
Creatinine	-2.39E^-03^	9.76E^-04^	0.01	9.99E^-03^	4.80E^-03^	0.04	-2.97E^-03^	1.54E^-03^	0.05	1.31E^-02^	7.47E^-03^	0.08
Creatinine'	1.64E^-06^	4.41E^-07^	<.01	-	-	-	2.37E^-06^	9.18E^-07^	0.01	-	-	-
eGFR(crea)	-3.48E^-03^	8.03E^-04^	<.01	-7.69E^-03^	3.78E^-03^	0.04	-2.42E^-03^	1.22E^-03^	0.05	-7.02E^-03^	5.16E^-03^	0.17
eGFR(crea)'	1.12E^-06^	2.40E^-07^	<.01	-	-	-	9.51E^-07^	4.22E^-07^	0.02	-	-	-
Cystatin C	0.25	0.12	0.03	1.18	0.54	0.03	0.05	0.20	0.81	2.53	0.90	<.01
Cystatin C'	1.67	0.86	0.05	-	-	-	5.81	2.27	0.01	-	-	-
eGFR(cys)	-4.79E^-03^	4.99E^-04^	<.01	-1.20E^-02^	5.53E^-03^	0.03	-7.76E^-03^	1.29E^-03^	<.01	-2.50E^-02^	9.19E^-03^	0.01
eGFR(cys)'	-	-	-	-	-	-	1.45E^-06^	5.98E^-07^	0.02	-	-	-
log(uACR)	5.47E-02	7.10E-03	<.01	-0.60	0.22	<.01	6.46E^-02^	1.26E^-02^	<.01	-1.01	0.39	<.01
log(uACR)'	-	-	-	9.52E^-02^	3.62E^-02^	<.01	-	-	-	0.23	0.09	0.01

eGFR = estimated glomerular filtration rate based on either serum creatinine [eGFR(crea)] or serum cystatin C concentration [eGFR(cys)]. *Models were adjusted for age, sex, waist circumference, smoking, total cholesterol, systolic and diastolic blood pressure and additionally in the whole population for diabetes mellitus type 2 and use of antihypertensive or antidiabetic medication. Creatinine', eGFR' and cystatin C' represent spline components, for more detail see method section.
