# Supplementary material for: Circulating Angiopoietin-2 and Its Soluble Receptor Tie-2 Concentrations Are Related to Renal Function in Two Population-Based Cohorts
Source: PLoS One. 2016 Nov 28;11(11):e0166492. doi: 10.1371/journal.pone.0166492 (PMC5125582; doi:10.1371/journal.pone.0166492)
Supplement: S1 File — Figure A. Scatterplot of creatinine-based [eGFR(crea)] versus cystatin C-based estimated glomerular filtration rate [eGFR(cys)] in both populations. Pearson correlation is given. (Figure A in S1 File) Figure B. Associations between serum creatinine concentration, creatinine-based estimated glomerular filtration rate [eGFR(crea)], serum cystatin C concentration or cystatin C-based eGFR [eGFR(cys)] and serum Tie-2 receptor concentration in the SHIP-1 (upper part) and SHIP-Trend (lower part) population. For each exposure left side: Estimated mean serum Tie-2 receptor with 95% confidence intervals (CI) by sex-specific quartiles of exposure calculated by analysis of variance adjusted for age, sex and waist circumference. Right side: linear regression line with 95% CI (grey shaded area). Linear regression models with restricted cubic splines were adjusted for age, sex, waist circumference, smoking, total cholesterol, systolic and diastolic blood pressure and additionally in the whole population for diabetes mellitus type 2 and use of antihypertensive medication. (Figure B in S1 File) Figure C. Associations between urinary albumin-to-creatinine ratio (uACR) and serum Tie-2 receptor concentration in SHIP-1 (left side) and SHIP-Trend (right side). For each study population left side: Estimated mean serum Tie-2 receptor with 95% confidence intervals (CI) by sex-specific quartiles of uACR calculated by analysis of variance adjusted for age, sex and waist circumference. Right side: linear regression line with 95% CI (grey shaded area). Linear regression models with restricted cubic splines were adjusted for age, sex, waist circumference, smoking, total cholesterol, systolic and diastolic blood pressure and additionally in the whole population for diabetes mellitus type 2 and use of antihypertensive medication. (Figure C in S1 File) (PDF) [file pone.0166492.s001.pdf]

S1 file

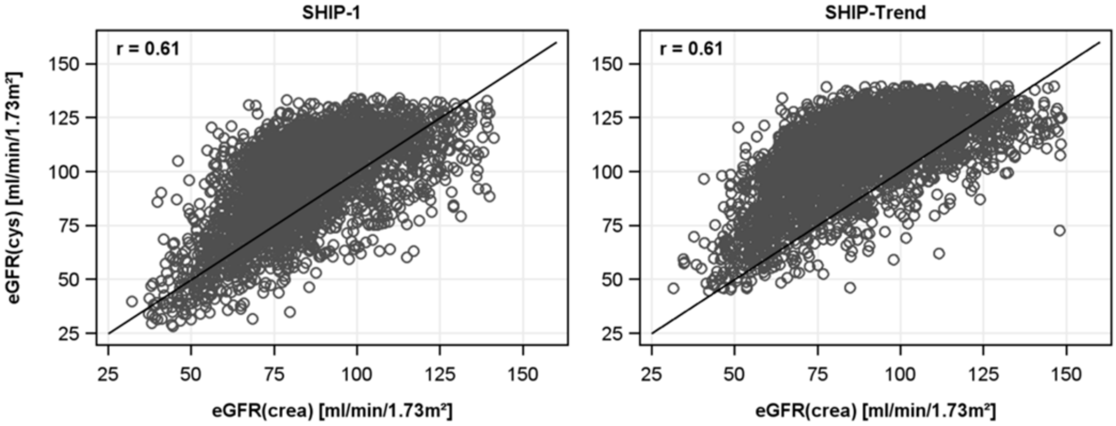

***Figure A*. Scatterplot of creatinine-based [eGFR(crea)] versus cystatin C-based estimated glomerular filtration rate [eGFR(cys)]** in both populations. Pearson correlation is given.

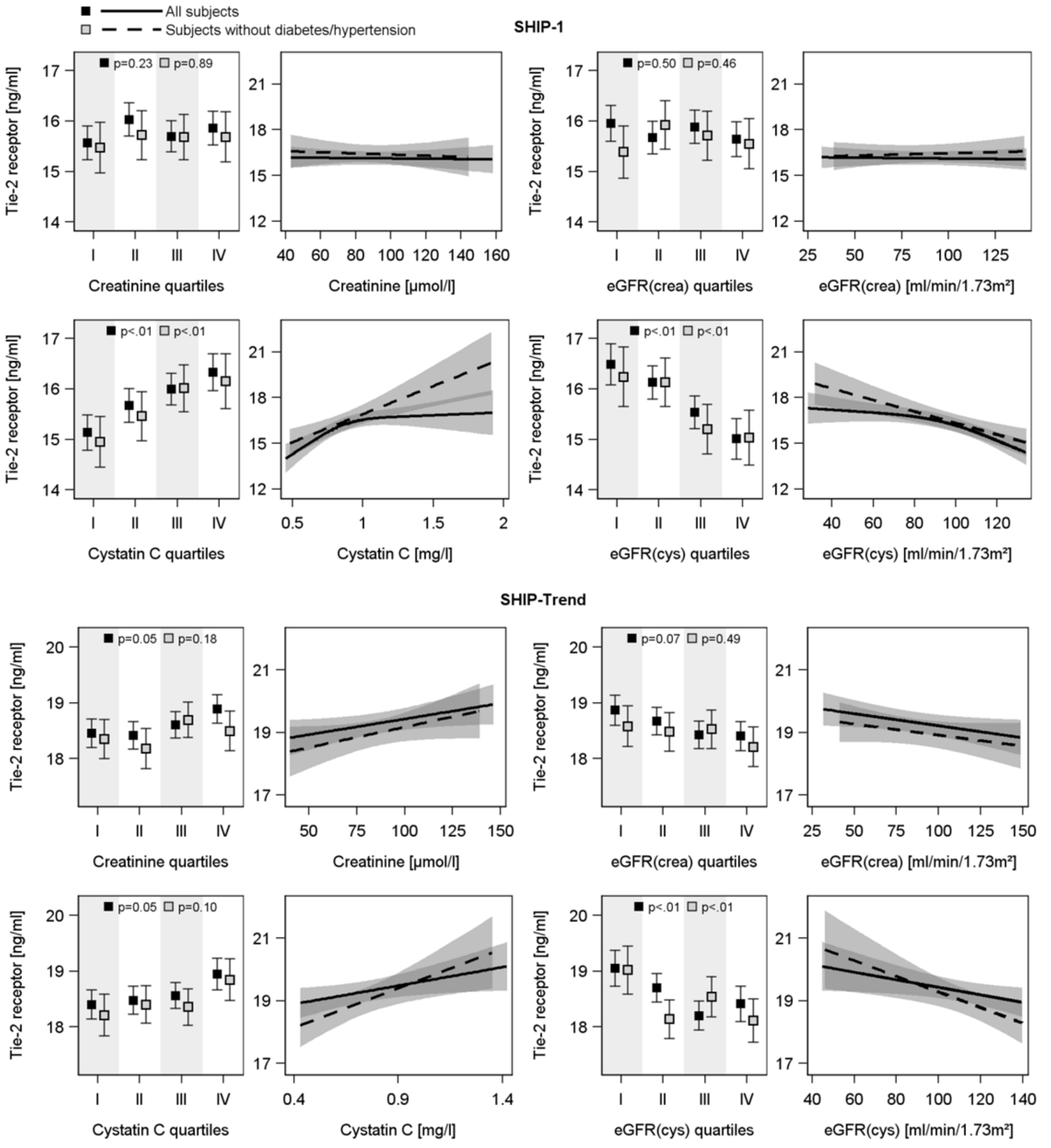

***Figure B*. Associations between serum creatinine concentration, creatinine-based estimated glomerular filtration rate [eGFR(crea)], serum cystatin C concentration or cystatin C-based eGFR [eGFR(cys)] and serum Tie-2 receptor concentration** in the SHIP-1 (upper part) and SHIP-Trend (lower part) population. For each exposure left side: Estimated mean serum Tie-2 receptor with 95% confidence intervals (CI) by sex-specific quartiles of exposure calculated by analysis of variance adjusted for age, sex and waist circumference. Right side: linear regression line with 95% CI (grey shaded area). Linear regression models with restricted cubic splines were adjusted for age, sex, waist circumference, smoking, total cholesterol, systolic and diastolic blood pressure and additionally in the whole population for diabetes mellitus type 2 and use of antihypertensive medication.

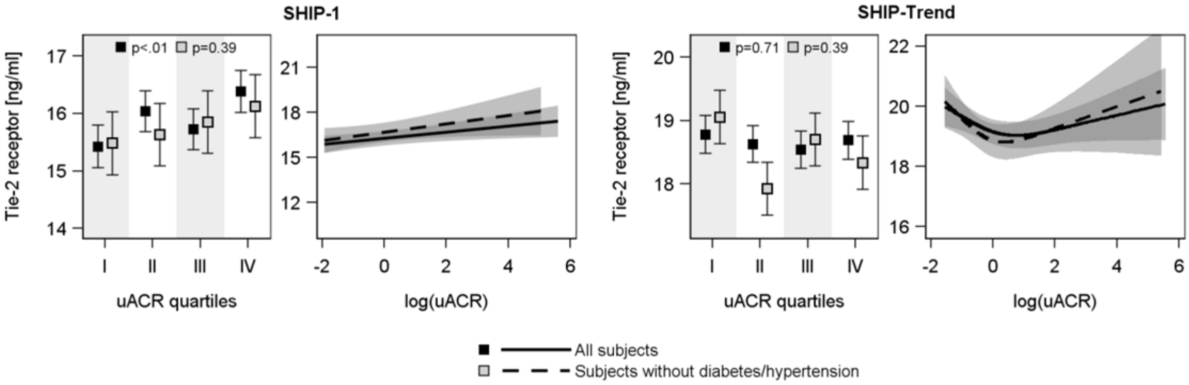

***Figure C*. Associations between urinary albumin-to-creatinine ratio (uACR) and serum Tie-2 receptor concentration** in SHIP-1 (left side) and SHIP-Trend (right side). For each study population left side: Estimated mean serum Tie-2 receptor with 95% confidence intervals (CI) by sex-specific quartiles of uACR calculated by analysis of variance adjusted for age, sex and waist circumference. Right side: linear regression line with 95% CI (grey shaded area). Linear regression models with restricted cubic splines were adjusted for age, sex, waist circumference, smoking, total cholesterol, systolic and diastolic blood pressure and additionally in the whole population for diabetes mellitus type 2 and use of antihypertensive medication.
